# Analysis of Lung Imaging Intelligent Diagnosis System for Nursing Intervention of Lung Cancer Patients' Quality of Life

**DOI:** 10.1155/2021/6750934

**Published:** 2021-11-15

**Authors:** Ruxia Guo, Hui Wang

**Affiliations:** Oncology Department Zhejiang Hospital, Hangzhou, Zhejiang 310030, China

## Abstract

In order to explore the influence of intelligent imaging diagnosis systems on comprehensive nursing intervention for patients with late-stage lung cancer, the system uses ITK and VTK toolkit to realize image reading, display, image marking, and interactive functions. The optimal threshold method and regional connectivity algorithm were used to segment the lung region, and then, the cavity filling algorithm and repair algorithm were used to repair the lung region. A variable ring filter was used to detect suspected shadows in the lungs. Finally, the classifier proposed in this paper is used to classify benign and malignant. The system has good sensitivity by detecting the images of real patients. 100 patients with advanced lung cancer were randomly divided into control group and nursing intervention group 50 cases each. Patients in the control group received routine radiotherapy and chemotherapy and routine nursing intervention. Patients in the nursing intervention group were given comprehensive nursing intervention on the basis of routine intervention in the control group for 2 consecutive months. Pittsburgh sleep quality index, pain degree, quality of life, and complications after intervention were compared between the 2 groups before and after intervention. The experimental results showed that the sleep quality, pain degree, quality of life, and complications in 2 groups were significantly improved after intervention (*P* < 0.05), and the improvement degree in the nursing intervention group was higher than that in the control group (*P* < 0.05). It is proved that comprehensive nursing intervention has a good effect on improving sleep quality, relieving physical pain, improving the quality of life, and reducing complications of lung cancer patients and can effectively improve the quality of life of lung cancer patients.

## 1. Introduction

Cancer has become a worldwide public health problem, attracting worldwide attention due to its high morbidity and mortality [1]. On February 4, 2011, ACS (American Cancer Society) published a report on global cancer statistics [2]. The report shows that the global burden of cancer is increasing due to age and population growth [3]. There were 12.7 million new cancer cases and 7.6 million cancer deaths in 2008, with 56 percent of the new cases and 64 percent of the deaths occurring in developing countries. Breast cancer is the most common cancer that causes new cancer cases and deaths in women, accounting for 23% of new cancer cases and 14% of cancer deaths, respectively [4]. Lung cancer is the most common cancer among men, accounting for 17% of new cancer cases and 23% of cancer deaths, respectively [5]. In January 2012, ACS released projected cancer data for 2012 in the United States. In 2012, there will be 1,638,910 new cancer cases and 577,190 deaths in the United States, an average of more than 1,500 deaths per day. Lung cancer accounted for 14 percent of all new cancer cases, the second most. Among all cancer deaths, lung cancer accounts for the highest proportion, accounting for 29% and 26% of male and female cancer deaths, respectively [6]. In cancer registration areas, lung cancer ranks first among all cancers with a mortality rate of 43.48/100,000, accounting for 25.3% of all cancer deaths, while in urban areas, the lung cancer mortality rate is 46.85/100,000, accounting for 28.16% of all cancer deaths [7]. It can be seen that lung cancer has become the number one killer of cancer, which is why more and more experts and scholars begin to conduct in-depth studies on the diagnosis and treatment of lung cancer [8].

According to statistics, the overall rate of lung cancer patients with no recurrence for more than 5 years after treatment is less than 15%, but the rate of early lung cancer patients with no recurrence for more than 5 years after treatment is up to 60%–90%, but the rate of early lung cancer is timely diagnosed is only 15% [9]. Early lung cancer symptoms are not obvious; it is difficult to detect; until the discovery, often to the terminal, cancer has metastasized. Therefore, early and timely detection of lung cancer is often more important than taking measures to cure it [10].

With the rapid development of the social economy, people's living standards have been greatly improved, but due to the fast pace of life and heavy life pressure, the incidence of lung cancer has increased [11]. Therefore, my internal and external studies continue. Farajpour et al. explored the clinical application significance of nursing intervention in patients with intermediate and advanced lung cancer. A total of 104 patients with advanced lung cancer admitted to PLA General Hospital from November 2011 to November 2012 were randomly divided into intervention group and control group, with 52 patients in each group. The two groups of patients with advanced lung cancer were given general routine nursing intervention, while the intervention group was added with a systematic nursing intervention program on this basis, and the quality of life and psychological state of the two groups of patients with advanced lung cancer were compared and analyzed in depth and detail [12]. Yuling et al. evaluated the effect of evidence-based nursing intervention on the quality of life and complication rate of patients with advanced lung cancer. 76 patients with advanced lung cancer in our hospital were divided into two groups, 38 patients in the control group and 38 patients in the study group. Routine nursing intervention and evidence-based nursing intervention were, respectively, applied to compare the quality of life and the incidence of complications between the two groups. After the intervention, the scores of physical vitality, mental state, physiological function, and social function of patients in the study group were significantly higher than those in the control group, and the incidence of complications was significantly lower than that in the control group (*P* < 0.05). The evidence-based nursing intervention has a positive impact on the quality of life and complication rate of patients with advanced lung cancer, which can effectively improve the quality of life of patients and timely prevent multiple complications [13]. Sw et al. showed the effect of nursing intervention on the quality of life of patients with advanced lung cancer. It was found that nursing intervention can effectively improve the quality of life of patients with advanced lung cancer, improve their anxiety, depression, and other adverse emotions, and relieve the fatigue state of patients, which is worthy of clinical application [14].

In this regard, the clinical nursing data of 100 patients with advanced lung cancer treated in this paper were summarized, and the conventional nursing and comprehensive nursing intervention of 100 patients with advanced lung cancer were compared and analyzed, and the report is as follows. In this regard, the clinical nursing data of 100 patients with advanced lung cancer treated in this paper were summarized, and the conventional nursing and comprehensive nursing intervention of 100 patients with advanced lung cancer were compared and analyzed, and the report is as follows.

## 2. Data and Methods

### 2.1. Subjects

A total of 100 patients diagnosed with advanced lung cancer in the Second Affiliated Hospital of a University of Traditional Chinese Medicine from July 2019 to January 2020 were divided into nursing intervention group and control group with 50 patients in each group by random number table method. In the nursing intervention group, there were 22 males and 28 females. Age ranged from 45 to 74 years, with an average of 55.33 ± 16.41 years. The course of the disease was 2–9 years, with an average of 2.7 ± 0.5 years. Pathological types were small cell carcinoma (7 cases), squamous cell carcinoma (25 cases), and adenocarcinoma (18 cases). In the control group, there were 29 males and 21 females. The average age ranged from 46 to 75, with 54.28 ± 15.37 years. The course of the disease was 2–8 years, with an average of 2.8 ± 0.6 years. Pathological types were 8 cases of small cell carcinoma, 20 cases of squamous cell carcinoma, and 22 cases of adenocarcinoma [15, 16]. There was no significant difference in baseline data between the two groups (*P* > 0.05), indicating comparability [17].

### 2.2. Tools Required for System Development

In order to achieve a fully functional and robust auxiliary diagnosis system for pulmonary nodules, the following two criteria should be followed. (1) The object-oriented approach is adopted to write different image processing algorithms in the form of classes, which is convenient for instantiation, code reuse, and subsequent function expansion [18, 19]. (2) Try to use existing and mature algorithms, software codes, or controls to make the system performance more stable. Based on the above ideas, the algorithm of the pulmonary nodules auxiliary diagnosis system uses the ITK class library to realize the processing of medical images. VTK library is used for 3d visualization of medical images to meet the needs of image display and interaction. In the mainstream Windows operating system, MFC is used to develop the user interface [20].

VTK is an object-oriented, open-source data visualization toolkit developed by Will Schroeder and others in C++. VTK consists of C++ class libraries and several interpretive interface layers such as Tcl/TK, Java, and Python. It can automatically convert C++ cores to Python, Java, and Tcl and can also write VTK applications in interpreted programming languages. VTK provides cross-platform development, supports a variety of operating systems, and also supports vector, vector, tensor, texture, and other algorithms.

Insight Segmentation and Registration Toolkit (ITK) is derived from The Visual Human Project of The National Library of Medicine of The United States of America and is a toolkit specially used for medical image segmentation and registration. ITK is open source to the public and supports cross-platform operation, providing mature and efficient image segmentation and registration algorithms, but not limited to this. ITK supports the C++ standard and is developed based on templates and generic programming technologies. It can easily expand the data types and dimensions of various classes in ITK, greatly enhancing the functions of ITK.

ITK and VTK functions have different focuses; therefore, the combination of ITK and VTK, learn from each other, to solve the ITK data processing function is strong but cannot be visualized, and VTK is not suitable for processing a large amount of data. Because ITK and VTK implementation methods are different, VTK uses the traditional object-oriented programming, and ITK is based on template and paradigm programming, ITK data cannot be directly used by VTK. ITK to VTK image filter class for us was used to achieve ITK data to VTK data conversion work, and ITK and VTK image processing pipelines are connected. ITK is responsible for image processing and VTK is responsible for image data display and interaction. ITK and VTK are software development packages themselves, and neither provides a graphical user interface (GUI). Graphical user interfaces including MFC, QT, and FLTK can be used to provide a good, user-friendly program interface and convenient user use. In the system development, in line with the principle of MFC widely used, we choose MFC to develop user interface.

### 2.3. Intelligent Pulmonary Imaging Diagnosis System

Through the study of a radiologist diagnosing pulmonary nodules, the CAD system of pulmonary nodules diagnosis is mainly divided into four steps. The first step is the segmentation of double lung regions, and the accurate range of lung is found in the chest CT image. The second step is the detection process of suspected pulmonary nodules. Suspected pulmonary nodules are found in the range of lungs found in the first step. The third step is the process of image feature extraction of suspected pulmonary nodules as the basis for further analysis. The fourth step is the discrimination process of suspected pulmonary nodules. Benign or malignant pulmonary nodules can be distinguished by the previously extracted features.

For a complete auxiliary diagnostic system, in addition to these four diagnostic processes, it should also include CT image reading, image display, and the marking and interaction of pulmonary nodules, so as to facilitate doctors to mark discovered pulmonary nodules. The whole system is divided into 7 parts, which are CT image reading, lung region segmentation, detection of suspected lung shadow, feature extraction of suspected lung shadow, discrimination of suspected lung shadow property, CT image display, and marking and interaction of suspected lung nodules. Among them, the middle of the four parts is the key to the system; each part of the effect of good or bad affects the performance of the whole system.

### 2.4. Nursing Methods

#### 2.4.1. Control Group

The conventional nursing intervention was used on the basis of conventional radiotherapy and chemotherapy. The nurses in charge of the bed are mainly responsible for routine health counseling and distributing health knowledge manuals for patients and their families to read and understand by themselves. We inform the patient's family members of the patient's condition before radiotherapy and chemotherapy, carry out popular science education, and actively cooperate with and assist medical staff in treatment. After the end of radiotherapy and chemotherapy, patients were observed for any adverse reactions.

#### 2.4.2. Nursing Intervention Group

On the basis of nursing intervention in the control group, a comprehensive nursing intervention was given. The nursing intervention methods were divided into the understanding of basic knowledge of the disease, the formulation of targeted diet prescription, psychological counseling nursing, pain nursing, and complication nursing guidance.

#### 2.4.3. Understanding of Basic Knowledge of Diseases

Based on the Cancer Nursing Manual, a large number of literature studies were consulted to write the Health Education Manual for lung cancer patients, and the basic concepts, diagnostic criteria, clinical symptoms, common treatment methods, and prognosis of lung cancer were popularized according to the contents of the education manual.

#### 2.4.4. Formulation of Dietary Prescriptions

Due to the serious harm and influence of radiotherapy and chemotherapy on the body of patients and the physical weakness of patients with radiotherapy and chemotherapy, dietary prescriptions should be formulated according to the principle of supplementing the benefits of deficiency in traditional Chinese medicine, according to the five flavors of traditional Chinese medicine, and properly mix the white food into the lung meridian to nourish the lung QI and the red food into the heart meridian to nourish the heart, such as astragalus stewed black chicken soup, red jujube boiled eggs, and cordyceps Sinensis soup. We establish a reasonable dietary nutritional structure, minimize the intake of animal offal, fried food, and high-salt and high-fat food, and increase the intake of vegetables, crude fiber, and protein.

#### 2.4.5. Psychological Counseling Nursing

Nursing for patients should be with patience to solve the problem which is put forward, through the conversation understanding. The psychological condition of the patients should be discussed in a timely manner to eliminate the fear of lung cancer patients with a psychological problem. There should be positive good confidence that she would win the battling fighting against cancer. Lung cancer patients generally have poor sleep quality. Through psychological counseling of patients, patients can build a good night's sleep structure, thus improving their quality of life.

#### 2.4.6. Nursing of Complications

Radiation and chemotherapy can cause serious adverse reactions, such as hair loss and vomiting. For patients with alopecia, nursing staff at the initial stage of alopecia carry out head massages to improve local circulation to reduce the number of alopecia and for severe alopecia as far as possible to reduce the patient's psychological pressure. They can ask the patient's family to buy comfortable wigs. For patients with vomiting, caregivers may give mints to remove odors from the mouth. The intervention lasted for 2 months in both groups.

### 2.5. Observation Indicators

#### 2.5.1. Pittsburgh Sleep Quality Index (PSQI)

PSQI was initiated by Buysee et al. in 1989, with seven portfolios of PSQI by 18 items; each project ranges from 0 to 3 points. Out of 21 points, the lower the score, the better the patient's quality of sleep, with seven points as the critical standard of judging sleep quality is normal. If the total score is greater than 7 points, this means that the patient's sleep quality is bad; an overall score of less than 7 indicates good sleep quality.

#### 2.5.2. Complications

The incidence of heart failure, respiratory failure, and arrhythmia in 2 groups was observed.

### 2.6. Statistical Methods

SPSS 13.5 was used for statistical analysis. The measurement data were expressed as (*x* ± *s*), *t*-test was used, and the count data was *x*^2^test. *P* < 0.05 was considered statistically significant.

## 3. The Results

### 3.1. System Test Results

**Figure 1:**
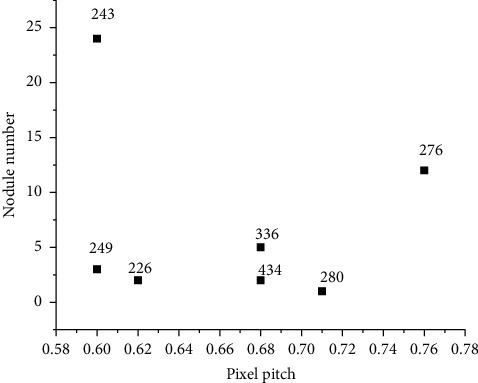
Patient image information.

**Figure 2:**
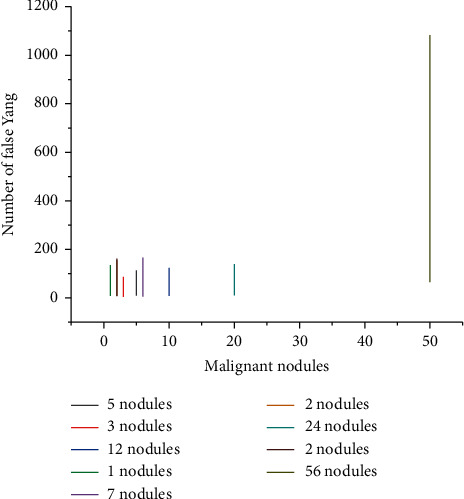
Detection results.

The system detected 8 sets of lung CT images of patients. The CT image details of different patients are shown in Figure 1. The number of CT images varies from patient to patient; the device manufacturer, pixel spacing, and layer spacing are also different. The system first reads the 3d image of the patient, then performs segmentation of the lung area, detects the suspected shadow of the segmented lung with variable ring filter, calculates the eigenvalue of the detected shadow, and finally classifies the suspected shadow by classifier to complete the whole assisted diagnosis process of pulmonary nodules. The overall detection results are shown in Figure 2. There were 2316 CT images with 56 nodules in 8 sets of different patient images. The system detected 49 nodules and missed 7, with a detection rate of 87.5%. A total of 65 false-positive nodules were detected, with an average of 8 nodules per set of images. Experimental results show that the system is effective for images of different devices, different pixel spacing, and layer spacing, with high overall sensitivity, and can provide effective advice for doctors. But there is still the problem of a high false number rate. Finally, 8 sets of CT images were detected by the system, and the detection rate was 87.5%. It can effectively provide good advice for doctors.

### 3.2. PSQI

PSQI scores of the two groups before and after intervention are shown in Table 1.

### 3.3. Occurrence of Complications

The incidence of complications in the two groups after the intervention is shown in Table 2.

## 4. Discussion

### 4.1. Relationship between PSQI Score and Sleep Disorders in Lung Cancer Patients

Sleep disorder is an important indicator to judge the physical condition of patients with lung cancer. Patients often have gastrointestinal dysfunction, body fatigue, hair loss, and adverse emotional reactions in the treatment process. These factors seriously affect the normal sleep of patients, and adequate sleep time is one of the important prerequisites for improving human immunity. The results of this study showed that PSQI scores in the two groups were lower after intervention than before, and PSQI scores in the nursing intervention group decreased more significantly than those in the control group, with statistically significant differences (*P* < 0.05). It shows that comprehensive nursing intervention has a better effect on improving the sleep quality of lung cancer patients.

### 4.2. Relationship between Quality of Life Score and Quality of Life of Lung Cancer Patients

Studies have shown differences in living habits and cultural literacy in different countries and races, but EORTC QLQ-C30 has become one of the important means to judge the quality of life of lung cancer patients with its good reliability and validity. The results of this study showed that after the intervention, the physiological function and physical condition scores of patients in the two groups were higher than those before the intervention, and the score of the nursing intervention group increased more significantly than that of the control group; the difference was statistically significant (*P* < 0.05). The score of clinical symptoms was lower than before the intervention, and the score of the nursing intervention group decreased more significantly than the control group; the difference was statistically significant (*P* < 0.05). It shows that comprehensive nursing intervention has a good effect on improving the quality of life of lung cancer patients.

### 4.3. Relationship between Pain Degree and Lung Cancer

Pain often accompanies the whole course of lung cancer patients and seriously affects their physical and mental health. Therefore, the evaluation of pain in patients with lung cancer is particularly important. The advantage of NRS lies in its features of simple operation, safe use, painless process, and efficient results, which are easily accepted by the majority of patients. The results of this study showed that the scores of patients in both groups were lower than before the intervention, and the decrease of the nursing intervention group was more obvious than that of the control group; the difference was statistically significant (*P* < 0.05). It shows that nursing intervention can effectively relieve pain in lung cancer patients.

### 4.4. Occurrence of Complications

Lung cancer is extremely harmful to the patient's body, and if it is combined with other diseases, it is worse for the patient. Therefore, the control of complications is very important to control the progression of lung cancer. The results of this study showed that the number of patients with heart failure, respiratory failure, and arrhythmia in the intervention nursing intervention group was lower than that in the control group, and the difference was statistically significant (*P* < 0.05). It shows that comprehensive nursing intervention has a good effect on reducing the incidence of complications in patients with lung cancer.

### 4.5. Effects of Radiotherapy and Chemotherapy on Lung Cancer Patients

The early stage of lung cancer is not easy to find, is mostly found in the middle-late age, and lost the best opportunity to surgical treatment. Radiotherapy and chemotherapy become the main methods to relieve patients' pain as they have the most efficient killing effects on tumor cells, with good clinical efficacy, but they will cause serious damage to patients' bodies and minds.

## 5. Conclusion

In order to explore the influence of intelligent image diagnosis system on comprehensive nursing intervention of patients with advanced lung cancer, the system uses ITK and VTK toolkit to realize image reading, display, image marking, and interaction. The optimal threshold method and region connectivity algorithm are used to segment the lung region, and then the cavity filling algorithm and repair algorithm are used to repair the lung region. 120 patients with advanced lung cancer were randomly divided into control group and nursing intervention group, with 60 cases in each group. The control group received routine radiotherapy and chemotherapy and routine nursing intervention. The nursing intervention group received comprehensive nursing intervention on the basis of routine intervention in the control group for 2 months. Pittsburgh sleep quality index, pain degree, quality of life, and complications were compared between the two groups before and after intervention. The results showed that the sleep quality, pain degree, quality of life, and complications of the two groups were significantly improved after the intervention (*P* < 0.05), and the improvement degree of the nursing intervention group was higher than that of the control group (*P* < 0.05). In conclusion, comprehensive nursing intervention has a good effect on improving the sleep quality of lung cancer patients, alleviating physical pain, improving the quality of life, and reducing complications and can effectively improve the quality of life of lung cancer patients.

## Figures and Tables

**Table 1:** Comparison of PSQI scores between the two groups before and after intervention (*x* ± *s*).

Group	Example number	Time	Sleep quality	Sleep time	Sleep efficiency	Sleeping pills	Daytime function
Control group	50	Before intervention	2.12 ± 0.65	2.52 ± 0.64	2.66 ± 0.71	2.46 ± 0.50	2.48 ± 0.73
		After intervention	2.10 ± 6.26	2.23 ± 24.02	2.28 ± 13.04	2.22 ± 28.44	2.30 ± 18.79

*T*			0.21	0.68	0.44	0.47	0.35
*P*			0.04	0.04	0.04	0.04	0.04
Nursing intervention group	50	Before intervention	2.23 ± 0.51	2.42 ± 0.73	2.65 ± 0.84	2.57 ± 0.82	2.49 ± 0.70
		After intervention	1.34 ± 0.41	1.73 ± 0.40	1.29 ± 0.36	1.61 ± 0.52	1.49 ± 0.40
*T*			1.20	1.18	1.25	1.42	1.02
*P*			0.03	0.03	0.03	0.03	0.03

**Table 2:** Comparison of quality of life scores between the two groups before and after intervention (*x* ± *s*).

Group	Example number	Heart failure		Failure of respiration		Heart rate is normal		Total	
		Example number	%	Example number	%	Example number	%	Example number	%
Control group	50	3	5.0	2	3.3	5	8.3	10	16.7
Nursing intervention group	50	2	3.3	1	1.7	1	1.7	4	6.7
*χ*2		1.10	1.10	4.32	6.15				
*P*		0.03	0.03	0.02	0.01				

## Data Availability

The data used to support the findings of this study are available from the corresponding author upon request.

## References

[1] He Y., Camacho R. S., Soygazi H., Luo C. (2021). Attacking and defence pathways for intelligent medical diagnosis system (IMDS). *International Journal of Medical Informatics*.

[2] Li X., Zhang W., Ding Q., Sun J.-Q. (2020). Intelligent rotating machinery fault diagnosis based on deep learning using data augmentation. *Journal of Intelligent Manufacturing*.

[3] Behera S. K., Parhi D. R., Das H. C. (2019). Approach to establish a hybrid intelligent model for crack diagnosis in a fix-hinge beam structure. *International Journal of Structural Integrity*.

[4] Wang J.-G., Zhou L.-B. (2019). Traffic light recognition with high dynamic range imaging and deep learning. *IEEE Transactions on Intelligent Transportation Systems*.

[5] Liang H., Zou J., Liang W. (2019). An early intelligent diagnosis model for drilling overflow based on GA–BP algorithm. *Cluster Computing*.

[6] Mallikarjuna P. B., Sreenatha M., Manjunath S., Kundur N. C. (2020). Aircraft gearbox fault diagnosis system: an approach based on deep learning techniques. *Journal of Intelligent Systems*.

[7] Wang M., Zhang Z., Li K., Zhang Z., Sheng Y., Liu S. (2020). Research on key technologies of fault diagnosis and early warning for high-end equipment based on intelligent manufacturing and internet of things. *International Journal of Advanced Manufacturing Technology*.

[8] Lin T., Liu X. (2021). An intelligent recognition system for insulator string defects based on dimension correction and optimized faster R-CNN. *Electrical Engineering*.

[9] Jia G., Han G., Du J., Chan S. (2019). PMS: intelligent pollution monitoring system based on the industrial internet of things for a healthier city. *IEEE Network*.

[10] Wang Z., Cheng Y., Li J., Hu X. (2021). Effect of integrated medical and nursing intervention model on quality of life and unhealthy emotion of patients with esophageal cancer undergoing radiotherapy. *American Journal of Tourism Research*.

[11] Hoseiniyan F., Amiri S., Bari M. R., Bari L. R., Dodangeh S. (2020). Effect of soy protein isolate and tio2 edible coating on quality and shelf-life of grapes varieties hosseini and ghezel ozom. *Food Science and Technology*.

[12] Farajpour P., Sheykhlouei H. (2021). Study on edible coating effect, based on aloevera gel and thymol on the postharvest quality and storage life of strawberry. *Food Science and Technology*.

[13] Yuling O., Guo S., Shiyuan G., Jieping C., Qiuqi L. (2019). Effect of food quality on life history traits of daphnia galeata and bosmina fatalis. *Journal of Lake Sciences*.

[14] Wiswell S., Bell J. G., McHale J., Elliott J. O., Rath K., Clements A. (2019). The effect of art therapy on the quality of life in patients with a gynecologic cancer receiving chemotherapy. *Gynecologic Oncology*.

[15] Tseng L.-C., Chen K.-H., Wang C.-L., Weng L.-C. (2020). Effects of tyrosine kinase inhibitor therapy on skin toxicity and skin-related quality of life in patients with lung cancer. *Medicine*.

[16] Sato N., Motoi F., Iseki M. (2019). Effect of neoadjuvant chemotherapy using gemcitabine and s1 before surgery for pancreatic cancer on quality of life. *Journal of Clinical Oncology*.

[17] Vinan-Vega M., Mantilla B., Yang S., Nugent K. (2021). The effect of pulmonary rehabilitation on physical performance and health related quality of life in patients with chronic lung disease. *Respiratory Medicine*.

[18] Cui Y., Li Y.-X. (2020). Effect of high-quality nursing on alleviating depression and anxiety in patients with thyroid cancer during perioperative period. *Medicine*.

[19] Choung R. S., Lamba A., Marietta E. V., See J. A., Murray J. A. (2019). Effect of a gluten-free diet on quality of life in patients with nonclassical versus classical presentations of celiac disease. *Journal of Clinical Gastroenterology*.

[20] Elfeki H., Larsen H. M., Emmertsen K. J. (2019). Bowel dysfunction after sigmoid resection for cancer and its impact on quality of life. *British Journal of Surgery*.

